# Supplementary material for: Investigating microbiota differences across chronic pancreatitis, influenced by lifestyle and genetic determinants
Source: BMC Gastroenterol. 2025 Oct 21;25:747. doi: 10.1186/s12876-025-04327-7 (PMC12541979; doi:10.1186/s12876-025-04327-7)
Supplement: Supplementary file 1 — Additional file 1. [file 12876_2025_4327_MOESM1_ESM.docx]

Investigating microbiota differences across chronic pancreatitis, influenced by lifestyle and genetic determinants

Abubaker Y.M Ahmed ^1^, Azita Rajai ^2,3^, Catherine Fullwood ^2,3^, Damian W Rivett ^4^, John McLaughlin ^5^, Christopher van der Gast ^6,7^, Ryan Marsh ^7, *^

^1^ Department of Gastroenterology, Manchester University NHS Foundation Trust, Manchester, United Kingdom

^2^ Research & Innovation, Manchester University NHS Foundation Trust, Manchester, United Kingdom

^3^ Centre for Biostatistics, University of Manchester, Manchester Academic Health Sciences Centre, Manchester, United Kingdom

^4^ Department of Natural Sciences, Manchester Metropolitan University, Manchester, United Kingdom

^5^ Faculty of Biology, Medicine and Health, Manchester Academic Health Sciences Centre, University of Manchester, Manchester, United Kingdom

^6^ Department of Respiratory Medicine, Northern Care Alliance NHS Foundation Trust, Salford, United Kingdom

^7^ Department of Applied Sciences, Northumbria University, Newcastle, United Kingdom

*** Corresponding Author**

*** Ryan3.marsh@northumbria.ac.uk**

**Additional File 1 – Supplementary Methods & Results**

**Supplementary Methods**

*Study participants and design*

Exclusion criteria for all participants included abstinence from prior antibiotic use within the previous 3 months, no diagnosis of alternate gastrointestinal conditions, abstinence from illicit drug use, and finally having no prior experience of diarrhoea lasting in excess of 48 hours within the last 6 months. All participants were asked to donate three stool samples using a home postal kit over the course of a 7-day period, which was met with relatively high adherence of 2.77 ± 0.55 (mean ± SD), to account for any intra-individual temporal variance. Upon receipt, samples were immediately stored at -80 °C at the Manchester Foundation Trust dedicated Biobank. Samples were individually processed and sequenced across both platforms. Additionally, basic participant clinical data was available for all groups, and is summarised in Table 1. Additional metadata, including specific pancreatic complications, surgical and endoscopic interventions, were reported by the consultant gastroenterologists following routine clinical practices.

*Predictive functional annotation using PICRUSt2*

To provide a more comprehensive understanding of microbial functional potential, identified KEGG orthologues (KO) were mapped to level 3 identified pathway. Additionally, identified enzyme commission (EC) numbers were linked to predicted functions. Results from both approaches were aggregated across samples for group-wise comparisons. This mapping was based on the KEGG pathway maps br08901 of BRITE Functional Hierarchies in the KEGG database (<http://www.genome.jp/kegg-bin/get_htext?br08901.keg>). The classification of enzyme functions was based on KEGG pathway map01100.keg from the BRITE Functional Hierarchies.

**Supplementary Results**

Following sequencing processing of the 16S rRNA gene data obtained from the Illumina MiSeq, a total of 4,704,612 reads were obtained, averaging (± SD) 49,522 ± 7,964 reads per sample. Full-length 16S rRNA sequencing on the PacBio Revio system yielded similar values, whereby a total of 3,123,278 reads were obtained, averaging (± SD) 36,744 ± 26,768 reads per sample. Samples with excessively low reads (<1000, n = 1) were removed from subsequent analyses due to unsuitability for subsequent similarity and diversity analyses.

| **Table S1** Individual metadata across the CP cohort. | | | | | | | | | |  |
| --- | --- | --- | --- | --- | --- | --- | --- | --- | --- | --- |
| **ID** | **Group** | **Endocrine insufficiency** | **Exocrine insufficiency** | **PERT** | **Endoscopic intervention** | **Cholecystectomy** | **Pancreatic complications** | **Smoker** | **Vitamin D (nmol)** |  |
| 016 | AIP | N | Y | Y | AD, ERCP (fistula closure) | N | PN | Y | 9.3 |  |
| 015 | AIP | Y | Y | Y | N | N | N | N | - |  |
| 017 | AIP | Y | Y | Y | ERCP (CBD stricture) | N | N | Y | 76.0 |  |
| 019 | AIP | Y | Y | Y | AD | N | PC | Y | - |  |
| 023 | AIP | N | Y | Y | AD | N | PC | Y | 73.0 |  |
| 018 | AIP | N | Y | Y | AD | N | PN | X | - |  |
| 020 | AIP | Y | Y | Y | None | N | N | N | - |  |
| 033 | AIP | Y | Y | Y | CB | N | N | Y | - |  |
| 031 | AIP | N | N | N | N | Y | PC | X | - |  |
| 028 | AIP | N | Y | Y | AD | Y | PN | Y | 117.0 |  |
| 030 | AIP | N | N | N | N | N | N | Y | 43.0 |  |
| 012 | CFRP | Y | Y | Y | ERCP | Y | CBD stones | N | - |  |
| 006 | CFRP | N | N | Y | N | N | N | N | 146.0 |  |
| 026 | CFRP | N | Y | Y | N | N | N | N | 113.0 |  |
| 034 | CFRP | N | Y | Y | N | N | N | N | 18.9 |  |
| 014 | CFRP | N | Y | Y | N | N | PN, PA | N | 67.8 |  |
| 008 | CFRP | N | N | N | N | N | N | N | - |  |
| 001 | CFRP | Y | Y | Y | N | N | N | X | 106.0 |  |
| 003 | CFRP | N | Y | Y | N | N | N | N | - |  |
| 007 | CFRP | N | N | N | N | Y | N | N | 72.0 |  |
| 010 | CFRP | Y | Y | Y | ERCP, DS | N | PDS | X | 62.5 |  |
| 011 | CFRP | N | N | N | N | N | N | N | - |  |
| 013 | CFRP | Y | Y | Y | N | N | N | N | 82.0 |  |
| 004 | CFRP | N | N | N | N | N | N | X | 16.0 |  |
| 009 | CFRP | Y | Y | Y | ERCP | Y | CBD stones | N | 102.0 |  |
| AIP; Alcohol-induced pancreatitis, AD; Axios drainage, CB; Coelic block, CBD; Common bile duct, CFRP; CFTR-related pancreatitis, DS; Pancreatic duct stent, ECRP; Endoscopic retrograde cholangiopancreatography, PA; Pseudoaneurysm, PC; Pseudocyst, PDS; Pancreatic distal stricture, PN; Pancreas necrosis, X; Ex-smoker. Y; Yes, N; No. Absent data is denoted with '-'. | | | | | | | | | |  |
|  |  |  |  |  |  |  |  |  |  |  |
|  |  |  |  |  |  |  |  |  |  |  |

|  | **Table S2** Goodness-of-fit summary statistics for core taxa against the log-normal distribution at ≥ 75% prevalence threshold. | | | | |  |
| --- | --- | --- | --- | --- | --- | --- |
|  |  |  |  |  |  |  |
|  |  | **FL-16S** | | **V4-16S** | |  |
|  | **Group** | *χ2* | *P (same)* | *χ2* | *P (same)* |  |
|  | Healthy controls | 3.349 | 0.851 | 5.652 | 0.581 |  |
|  | Alcohol-induced pancreatitis | 8.398 | 0.210 | 3.095 | 0.542 |  |
|  | CFTR-related pancreatitis | 8.562 | 0.073 | 4.932 | 0.553 |  |
|  |  |  |  |  |  |  |
|  |  |  |  |  |  |  |

|  | **Table S3** Fisher’s alpha diversity summary statistics between groups across various microbiota partitions. | | | | | | |
| --- | --- | --- | --- | --- | --- | --- | --- |
|  |  |  |  |  |  |  |  |
|  |  | Microbiota | | Core taxa | | Satellite taxa | |
| FL-16S | Overall | KW-χ² | 5.0366 | KW-χ² | 1.2998 | KW-χ² | 5.1220 |
|  |  | *Df* | 2 | *df* | 2 | *df* | 2 |
|  |  | *p* (bonf) | 0.0806 | *p* (bonf) | 0.5221 | *p* (bonf) | 0.07723 |
|  | HC_AIP | *w* | 34 | *w* | 59 | *w* | 39 |
|  |  | *P* (bonf) | 0.261 | *P* (bonf) | 0.5027 | *P* (bonf) | 0.4561 |
|  | HC_CFRP | *w* | 78 | *w* | 74 | *w* | 82 |
|  |  | *P* (bonf) | 0.2093 | *P* (bonf) | 0.3237 | *P* (bonf) | 0.1264 |
|  | AIP_CFRP | *w* | 107 | *w* | 82 | *w* | 107 |
|  |  | *P* (bonf) | 0.04095 | *P* (bonf) | 0.5691 | *P* (bonf) | 0.04095 |
| V4-16S | Overall | KW-χ² | 2.2943 | KW-χ² | 26.8380 | KW-χ² | 4.7129 |
|  |  | *df* | 2 | *df* | 2 | *df* | 2 |
|  |  | *P* (bonf) | 0.3175 | *P* (bonf) | 0.0001> | *P* (bonf) | 0.09 |
|  | HC_AIP | *w* | 49 | *w* | 99 | *w* | 26 |
|  |  | *P* (bonf) | 1 | *P* (bonf) | 0.0001> | *P* (bonf) | 0.0804 |
|  | HC_CFRP | *w* | 79 | *w* | 125 | *w* | 70 |
|  |  | *P* (bonf) | 0.34 | *P* (bonf) | 0.0001> | *P* (bonf) | 0.69 |
|  | AIP_CFRP | *w* | 104 | *w* | 8 | *w* | 112 |
|  |  | *P* (bonf) | 0.15 | *P* (bonf) | 0.0001> | *P* (bonf) | 0.06 |


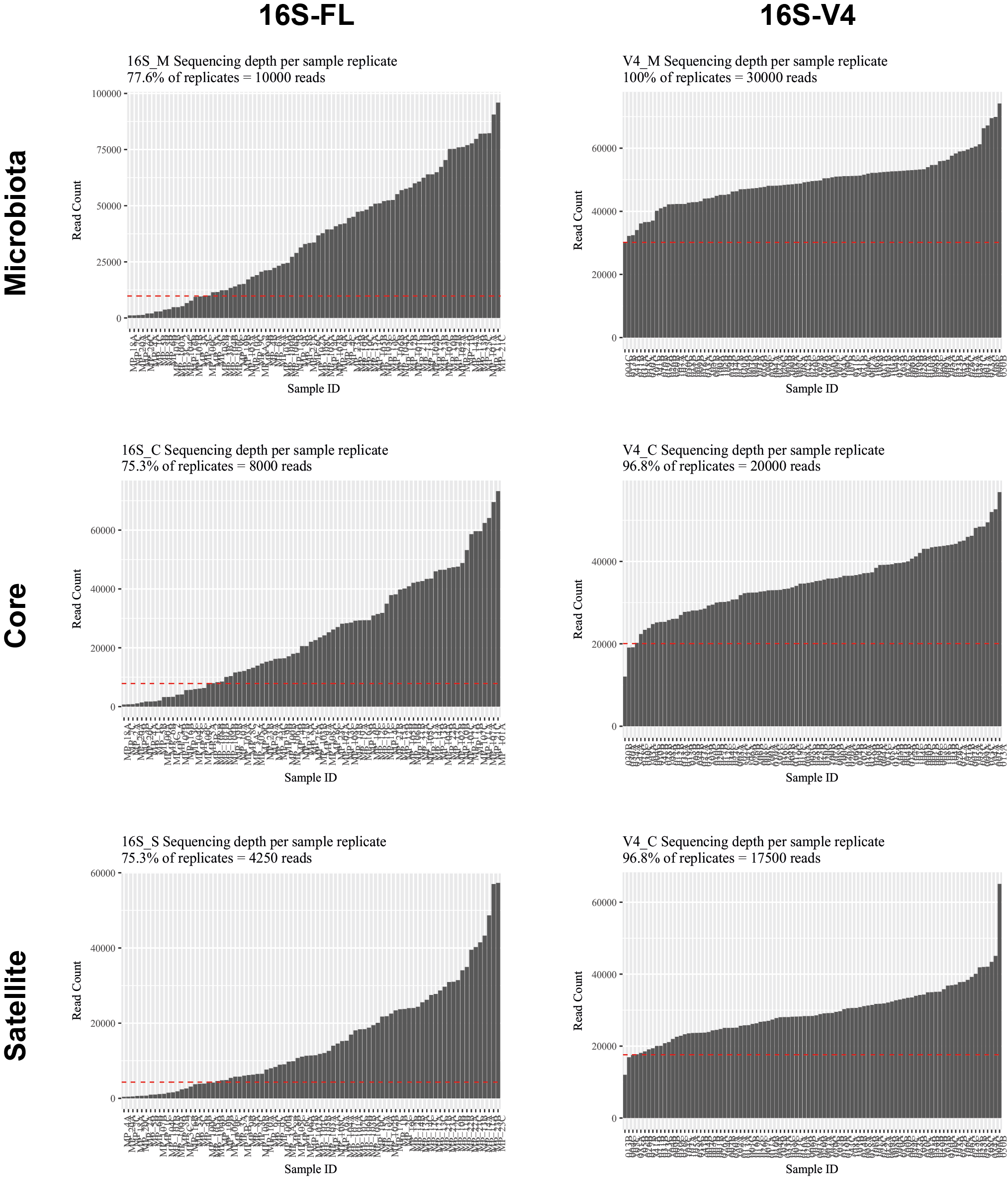


**Fig S1**. Sequencing depth across each partition of the microbial community across both the FL-16S and V4-16S datasets. Rarefaction depth was chosen to preserve maximal sample replicate data for each participant as to reduce potential loss of participants from subsequent diversity analyses. Red-dashed line represents optimal rarefaction depth in each partition and dataset. Participants retained following rarefaction:16S-FL; microbiota (88.2%), core (82.4%), satellite (85.3%), V4-16S; all partitions 100%.

|  | **Table S4** Shannon diversity summary statistics between groups across various microbiota partitions. | | | | | | |
| --- | --- | --- | --- | --- | --- | --- | --- |
|  |  |  |  |  |  |  |  |
|  |  | Microbiota | | Core taxa | | Satellite taxa | |
| FL-16S | Overall | KW-χ² | 1.4402 | KW-χ² | 1.3717 | KW-χ² | 0.0857 |
|  |  | *df* | 2 | *df* | 2 | *df* | 2 |
|  |  | *p* (bonf) | 1.000 | *p* (bonf) | 1.000 | *p* (bonf) | 1.000 |
|  | HC_AIP | *w* | 60 | *w* | 59 | *w* | 41 |
|  |  | *P* (bonf) | 0.729 | *P* (bonf) | 0.834 | *P* (bonf) | 1.000 |
|  | HC_CFRP | *w* | 50 | *w* | 50 | *w* | 50 |
|  |  | *P* (bonf) | 1.000 | *P* (bonf) | 1.000 | *P* (bonf) | 1.000 |
|  | AIP_CFRP | *w* | 67 | *w* | 40 | *w* | 49 |
|  |  | *P* (bonf) | 1.000 | *P* (bonf) | 1.000 | *P* (bonf) | 1.000 |
| V4-16S | Overall | KW-χ² | 1.5733 | KW-χ² | 0.2125 | KW-χ² | 3.0399 |
|  |  | *df* | 2 | *df* | 2 | *df* | 2 |
|  |  | *P* (bonf) | 1.000 | *P* (bonf) | 1.000 | *P* (bonf) | 1.000 |
|  | HC_AIP | *w* | 57 | *w* | 51 | *w* | 63 |
|  |  | *P* (bonf) | 1.000 | *P* (bonf) | 1.000 | *P* (bonf) | 0.993 |
|  | HC_CFRP | *w* | 49 | *w* | 54 | *w* | 53 |
|  |  | *P* (bonf) | 1.000 | *P* (bonf) | 1.000 | *P* (bonf) | 1.000 |
|  | AIP_CFRP | *w* | 97 | *w* | 81 | *w* | 108 |
|  |  | *P* (bonf) | 0.873 | *P* (bonf) | 1.000 | *P* (bonf) | 0.286 |

|  | **Table S5** ANOSIM summary statistics across both FL and V4 16S rRNA analyses of microbiota composition. | | | | | | |
| --- | --- | --- | --- | --- | --- | --- | --- |
|  |  |  |  |  |  |  |  |
|  |  | Microbiota | | Core taxa | | Satellite taxa | |
| FL-16S | HC_AIP | *R* | 0.1011 | *R* | 0.1628 | *R* | 0.4332 |
|  |  | *P (same)* | 0.0483 | *P (same)* | 0.0143 | *P (same)* | 0.0001 |
|  |  | *P* (bonf) | 0.0489 | *P* (bonf) | 0.0151 | *P* (bonf) | 0.0002 |
|  |  | Perm N | 9999 | Perm N | 9999 | Perm N | 9999 |
|  | HC_CFRP | *R* | -0.01335 | *R* | 0.05083 | *R* | 0.3321 |
|  |  | *P (same)* | 0.523 | *P (same)* | 0.207 | *P (same)* | 0.0001 |
|  |  | *P* (bonf) | 0.5272 | *P* (bonf) | 0.2084 | *P* (bonf) | 0.0002 |
|  |  | Perm N | 9999 | Perm N | 9999 | Perm N | 9999 |
|  | AIP_CFRP | *R* | 0.0499 | *R* | 0.1262 | *R* | 0.1984 |
|  |  | *P (same)* | 0.1343 | *P (same)* | 0.0181 | *P (same)* | 0.0018 |
|  |  | *P* (bonf) | 0.1369 | *P* (bonf) | 0.0187 | *P* (bonf) | 0.0028 |
|  |  | Perm N | 9999 | Perm N | 9999 | Perm N | 9999 |
| V4-16S | HC_AIP | *R* | 0.04762 | *R* | 0.5376 | *R* | 0.5553 |
|  |  | *P (same)* | 0.1628 | *P (same)* | 0.0001 | *P (same)* | 0.0001 |
|  |  | *P* (bonf) | 0.1666 | *P* (bonf) | 0.0001 | *P* (bonf) | 0.0001 |
|  |  | Perm N | 9999 | Perm N | 9999 | Perm N | 9999 |
|  | HC_CFRP | *R* | -0.06149 | *R* | 0.3441 | *R* | 0.5572 |
|  |  | *P (same)* | 0.782 | *P (same)* | 0.0002 | *P (same)* | 0.0001 |
|  |  | *P* (bonf) | 0.791 | *P* (bonf) | 0.0002 | *P* (bonf) | 0.0001 |
|  |  | Perm N | 9999 | Perm N | 9999 | Perm N | 9999 |
|  | AIP_CFRP | *R* | 0.01308 | *R* | 0.4217 | *R* | 0.2988 |
|  |  | *P (same)* | 0.3506 | *P (same)* | 0.0001 | *P (same)* | 0.0001 |
|  |  | *P* (bonf) | 0.3534 | *P* (bonf) | 0.0001 | *P* (bonf) | 0.0001 |
|  |  | Perm N | 9999 | Perm N | 9999 | Perm N | 9999 |

| **Table S6** Similarity of percentage (SIMPER) analysis of microbiota dissimilarity (Bray-Curtis) between study groups, utilising the full-length 16S rRNA sequencing data. | | | | | | | | |  |
| --- | --- | --- | --- | --- | --- | --- | --- | --- | --- |
|  |  |  |  |  |  |  |  |  |  |
| ***Healthy controls vs Alcohol induced pancreatitis (70.5%)*** | | | | | | **Kruskal-Wallis** | | |  |
| **Taxon** | **Mean HC** | **Mean AIP** | **Av. dissim** | **% contrib.** | **Cumulative %** | **KW-χ²** | ***p*** | ***p* (FDR)** |  |
| *Bifidobacterium adolescentis* | 9.48 | 2.67 | 3.91 | 5.54 | 5.54 | 6.097 | 0.014 | **0.045** |  |
| *Ruminococcus bromii* | 4.53 | 1.16 | 2.13 | 3.02 | 8.56 | 3.756 | 0.053 | 0.132 |  |
| *Faecalibacterium prausnitzii* | 6.08 | 2.95 | 2.03 | 2.88 | 11.44 | 6.097 | 0.014 | **0.045** |  |
| *Bifidobacterium longum* | 3.33 | 3 | 1.7 | 2.41 | 13.85 | 0.244 | 0.621 | 0.676 |  |
| *Prevotella sp900551275* | 2.97 | 0.65 | 1.64 | 2.32 | 16.17 | 1.156 | 0.282 | 0.436 |  |
| *Agathobacter rectalis* | 2.7 | 3.35 | 1.53 | 2.17 | 18.34 | 0.175 | 0.676 | 0.676 |  |
| *Akkermansia muciniphila* | 0.38 | 2.8 | 1.4 | 1.99 | 20.33 | 0.766 | 0.382 | 0.477 |  |
| *Ruminococcus E sp003526955* | 0.01> | 2.76 | 1.38 | 1.96 | 22.28 | 6.399 | 0.011 | **0.045** |  |
| *Dorea formicigenerans* | 3.6 | 2.68 | 1.33 | 1.89 | 24.17 | 1.571 | 0.210 | 0.420 |  |
| *Streptococcus pasteurianus* | 0.01> | 2.5 | 1.25 | 1.77 | 25.95 | 1.051 | 0.305 | 0.436 |  |
| ***Healthy controls vs CFTR-related pancreatitis (66.8%)*** | | | | | | **Kruskal-Wallis** | | |  |
| **Taxon** | **Mean HC** | **Mean CFRP** | **Av. dissim** | **% contrib.** | **Cumulative %** | **KW-χ²** | ***p*** | ***p* (FDR)** |  |
| *Bifidobacterium adolescentis* | 9.48 | 6.18 | 4.31 | 6.45 | 6.45 | 2.061 | 0.151 | 0.252 |  |
| *Faecalibacterium prausnitzii* | 6.08 | 2.52 | 2.11 | 3.16 | 9.62 | 8.438 | 0.004 | **0.018** |  |
| *Ruminococcus bromii* | 4.53 | 2.76 | 2.01 | 3.01 | 12.63 | 0.938 | 0.333 | 0.370 |  |
| *Streptococcus lutetiensis* | 0.01> | 3.17 | 1.58 | 2.37 | 15 | 5.111 | 0.024 | 0.079 |  |
| *Bifidobacterium longum* | 3.33 | 3.71 | 1.54 | 2.3 | 17.3 | 0.055 | 0.815 | 0.815 |  |
| *Prevotella sp900551275* | 2.97 | 0.03 | 1.49 | 2.23 | 19.53 | 1.743 | 0.187 | 0.267 |  |
| *Blautia A sp003471165* | 4.2 | 3.43 | 1.45 | 2.18 | 21.71 | 3.622 | 0.057 | 0.143 |  |
| *Romboutsia timonensis* | 2.79 | 1.96 | 1.3 | 1.95 | 23.65 | 1.142 | 0.285 | 0.356 |  |
| *Agathobacter rectalis* | 2.7 | 2.29 | 1.3 | 1.94 | 25.6 | 2.463 | 0.117 | 0.233 |  |
| *Streptococcus salivarius* | 0.171 | 2.49 | 1.18 | 1.77 | 27.36 | 8.438 | 0.004 | **0.018** |  |
| ***Alcohol induced pancreatitis vs CFTR-related pancreatitis (75.6%)*** | | | | | | **Kruskal-Wallis** | | |  |
| **Taxon** | **Mean AIP** | **Mean CFRP** | **Av. dissim** | **% contrib.** | **Cumulative %** | **KW-χ²** | ***p*** | ***p* (FDR)** |  |
| *Bifidobacterium adolescentis* | 2.67 | 6.18 | 3.16 | 4.24 | 4.24 | 0.215 | 0.643 | 0.917 |  |
| *Agathobacter rectalis* | 3.35 | 2.29 | 1.8 | 2.41 | 6.65 | 0.272 | 0.602 | 0.917 |  |
| *Bifidobacterium longum* | 3 | 3.71 | 1.77 | 2.38 | 9.03 | 1.411 | 0.235 | 0.645 |  |
| *Streptococcus lutetiensis* | 0.17 | 3.17 | 1.65 | 2.21 | 11.23 | 0.580 | 0.446 | 0.893 |  |
| *Blautia A sp003471165* | 3.04 | 3.43 | 1.52 | 2.04 | 13.27 | 0.102 | 0.750 | 0.917 |  |
| *Faecalibacterium prausnitzii* | 2.95 | 2.52 | 1.5 | 2.02 | 15.29 | 0.001 | 0.977 | 0.977 |  |
| *Akkermansia muciniphila* | 2.8 | 0.44 | 1.44 | 1.93 | 17.22 | 1.279 | 0.258 | 0.645 |  |
| *Ruminococcus E sp003526955* | 2.76 | 0.09 | 1.38 | 1.86 | 19.07 | 4.475 | 0.034 | 0.339 |  |
| *Streptococcus pasteurianus* | 2.5 | 0.01> | 1.25 | 1.68 | 20.75 | 0.049 | 0.825 | 0.917 |  |
| *Ruminococcus bromii* | 1.16 | 2.76 | 1.21 | 1.62 | 22.36 | 3.336 | 0.068 | 0.339 |  |
| Taxa identified as core are highlighted in orange, whereas satellite taxa are highlighted in grey. Mean relative abundance (%) is also provided for each group. Percentage contribution is the mean contribution divided by the mean dissimilarity across samples. Mean dissimilarity across comparative groups is indicated in each table header. Cumulative percent does not equal 100% as the list is not exhaustive. Taxon identification should be considered putative, given that some species cannot be fully resolved to the species level utilising the full length of the 16S rRNA gene. Following SIMPER analyses, Kruskal-Wallis testing between groups ensued, with FDR-adjusted (Benjamini-Hochberg) *P* values reported in-table. Significant *p* (FDR) values are highlighted in bold. | | | | | | | | |  |
|  |  |  |  |  |  |  |  |  |  |
|  |  |  |  |  |  |  |  |  |  |
|  |  |  |  |  |  |  |  |  |  |
|  |  |  |  |  |  |  |  |  |  |
|  |  |  |  |  |  |  |  |  |  |

| **Table S7** Top five taxa driving microbiota dissimilarity from similarity of percentage (SIMPER) analysis of microbiota dissimilarity (Bray-Curtis) between study groups, utilising the full-length 16S rRNA sequencing data. | | | | |  |
| --- | --- | --- | --- | --- | --- |
|  |  |  |  |  |  |
| **Taxon** | **Mean HC** | **Mean AIP** | **Mean CFRP** | **Mean contribution to dissimilarity observed across groups (%)** |  |
| *Bifidobacterium adolescentis* | 9.48 | 2.67 | 6.18 | 5.41 |  |
| *Faecalibacterium prausnitzii* | 6.08 | 2.95 | 2.52 | 2.69 |  |
| *Ruminococcus bromii* | 4.53 | 1.16 | 2.76 | 2.55 |  |
| *Bifidobacterium longum* | 3.33 | 3.00 | 3.71 | 2.36 |  |
| *Agathobacter rectalis* | 2.70 | 3.35 | 2.29 | 2.17 |  |
| All taxa are identified as core as denoted by orange cells. Mean relative abundance (%) for a given taxon is provided for each group. Mean dissimilarity of a given taxon across all comparator groups is indicated in the last column. Taxon identification should be considered putative, given that some species cannot be fully resolved to the species level utilising the full length of the 16S rRNA gene. Detailed between-group comparisons can be found in Table S6. | | | | |  |
|  |  |  |  |  |  |
|  |  |  |  |  |  |
|  |  |  |  |  |  |
|  |  |  |  |  |  |
|  |  |  |  |  |  |

| **Table S8** Similarity of percentage (SIMPER) analysis of microbiota dissimilarity (Bray-Curtis) between study groups, utilising the V4 16S rRNA sequencing data. | | | | | | | | |  |
| --- | --- | --- | --- | --- | --- | --- | --- | --- | --- |
|  |  |  |  |  |  |  |  |  |  |
| ***Healthy controls vs Alcohol induced pancreatitis (63.5%)*** | | | | | | **Kruskal-Wallis** | | |  |
| **Taxon** | **Mean HC** | **Mean AIP** | **Av. dissim** | **% Contrib.** | **Cumulative %** | **KW-χ²** | ***p*** | ***p* (FDR)** |  |
| *Escherichia coli* | 3.22 | 6.77 | 3.62 | 5.71 | 5.71 | 2.433 | 0.119 | 0.198 |  |
| *Bifidobacterium adolescentis* | 6.07 | 1.72 | 2.62 | 4.13 | 9.84 | 3.931 | 0.047 | 0.118 |  |
| *Prevotella_9 copri* | 4.6 | 1.02 | 2.51 | 3.96 | 13.79 | 1.720 | 0.190 | 0.271 |  |
| *Faecalibacterium prausnitzii* | 7.05 | 4.83 | 2.05 | 3.23 | 17.03 | 4.056 | 0.044 | 0.118 |  |
| *Ruminococcus bromii* | 4.02 | 1.58 | 1.85 | 2.91 | 19.94 | 2.709 | 0.100 | 0.198 |  |
| *Subdoligranulum variabile* | 3.18 | 2.94 | 1.52 | 2.39 | 22.33 | 0.418 | 0.518 | 0.619 |  |
| *Blautia massiliensis* | 4.48 | 2.08 | 1.42 | 2.24 | 24.56 | 5.027 | 0.025 | 0.118 |  |
| *Akkermansia muciniphila* | 0.52 | 2.81 | 1.41 | 2.23 | 26.79 | 0.248 | 0.619 | 0.619 |  |
| *Agathobacter rectalis* | 3.33 | 2.96 | 1.31 | 2.06 | 28.85 | 0.325 | 0.569 | 0.619 |  |
| *Ruminococcoides bili* | 0 | 2.54 | 1.27 | 2 | 30.85 | 6.396 | 0.011 | 0.114 |  |
| ***Healthy controls vs CFTR-related pancreatitis (59.5%)*** | | | | | | **Kruskal-Wallis** | | |  |
| **Taxon** | **Mean HC** | **Mean CFRP** | **Av. dissim** | **% Contrib.** | **Cumulative %** | **KW-χ²** | ***p*** | ***p* (FDR)** |  |
| *Escherichia coli* | 3.22 | 4.19 | 2.77 | 4.66 | 4.66 | 0.483 | 0.487 | 0.541 |  |
| *Bifidobacterium adolescentis* | 6.07 | 3.19 | 2.71 | 4.56 | 9.22 | 3.131 | 0.077 | 0.145 |  |
| *Prevotella_9 copri* | 4.6 | 0.16 | 2.33 | 3.91 | 13.12 | 2.932 | 0.087 | 0.145 |  |
| *Agathobacter rectalis* | 3.33 | 4.18 | 2 | 3.37 | 16.49 | 3.813 | 0.051 | 0.145 |  |
| *Ruminococcus bromii* | 4.02 | 2.65 | 1.82 | 3.05 | 19.54 | 0.727 | 0.394 | 0.516 |  |
| *Faecalibacterium prausnitzii* | 7.05 | 6.57 | 1.53 | 2.57 | 22.11 | 0.671 | 0.413 | 0.516 |  |
| *Subdoligranulum variabile* | 3.18 | 3.15 | 1.41 | 2.37 | 24.47 | 0.120 | 0.729 | 0.729 |  |
| *Blautia OTU 2* | 4.14 | 3.52 | 1.33 | 2.23 | 26.7 | 3.111 | 0.078 | 0.145 |  |
| *Faecalibacterium duncaniae* | 3.66 | 1.93 | 1.28 | 2.15 | 28.85 | 6.671 | 0.010 | 0.098 |  |
| *Catenibacterium mitsuokai* | 2.43 | 0 | 1.22 | 2.04 | 30.89 | 5.104 | 0.024 | 0.119 |  |
| ***Alcohol induced pancreatitis vs CFTR-related pancreatitis (66.4%)*** | | | | | | **Kruskal-Wallis** | | |  |
| **Taxon** | **Mean AIP** | **Mean CFRP** | **Av. dissim** | **% Contrib.** | **Cumulative %** | **KW-χ²** | ***p*** | ***p* (FDR)** |  |
| *Escherichia coli* | 6.77 | 4.19 | 3.79 | 5.71 | 5.71 | 1.386 | 0.239 | 0.485 |  |
| *Faecalibacterium prausnitzii* | 4.83 | 6.57 | 2.3 | 3.47 | 9.17 | 2.186 | 0.139 | 0.464 |  |
| *Agathobacter rectalis* | 2.96 | 4.18 | 2.25 | 3.39 | 12.56 | 0.300 | 0.584 | 0.826 |  |
| *Bifidobacterium adolescentis* | 1.72 | 3.19 | 1.79 | 2.69 | 15.25 | 0.052 | 0.819 | 0.827 |  |
| *Akkermansia muciniphila* | 2.81 | 1.18 | 1.65 | 2.49 | 17.73 | 0.531 | 0.466 | 0.777 |  |
| *Subdoligranulum variabile* | 2.94 | 3.15 | 1.64 | 2.47 | 20.21 | 0.192 | 0.661 | 0.826 |  |
| *Blautia OTU 2* | 3.45 | 3.52 | 1.49 | 2.24 | 22.44 | 0.048 | 0.827 | 0.827 |  |
| *Blautia massiliensis* | 2.08 | 3.97 | 1.36 | 2.04 | 24.48 | 3.265 | 0.071 | 0.354 |  |
| *Ruminococcus bromii* | 1.58 | 2.65 | 1.27 | 1.92 | 26.4 | 1.366 | 0.243 | 0.485 |  |
| *Ruminococcoides bili* | 2.54 | 0.09 | 1.27 | 1.92 | 28.31 | 6.365 | 0.012 | 0.116 |  |
| Taxa identified as core are highlighted in orange, whereas satellite taxa are highlighted in grey. Mean relative abundance (%) is also provided for each group. Percentage contribution is the mean contribution divided by the mean dissimilarity across samples. Mean dissimilarity across comparative groups is indicated in each table header. Cumulative percent does not equal 100% as the list is not exhaustive. Taxon identification should be considered putative, given that some species cannot be fully resolved to the species level utilising the V4 region of the 16S rRNA gene. Following SIMPER analyses, Kruskal-Wallis testing between groups ensued, with FDR-adjusted (Benjamini-Hochberg) *P* values reported in-table. Significant *p* (FDR) values are highlighted in bold. | | | | | | | | |  |
|  |  |  |  |  |  |  |  |  |  |
|  |  |  |  |  |  |  |  |  |  |
|  |  |  |  |  |  |  |  |  |  |
|  |  |  |  |  |  |  |  |  |  |
|  |  |  |  |  |  |  |  |  |  |

| **Table S9** ANOSIM summary statistics across relative pathway abundance for annotated KEGG level 3 hierarchical pathways across all three study groups, utilising whole microbiota composition. | | |  |
| --- | --- | --- | --- |
|  |  |  |  |
|  |  |  |  |
|  | Microbiota | |  |
| **HC_AIP** | *R* | 0.1116 |  |
|  | *P (same)* | 0.0587 |  |
|  | *P* (bonf) | 0.0597 |  |
|  | Perm N | 9999 |  |
| **HC_CFRP** | *R* | -0.01874 |  |
|  | *P (same)* | 0.5339 |  |
|  | *P* (bonf) | 0.5374 |  |
|  | Perm N | 9999 |  |
| **AIP_CFRP** | *R* | 0.03528 |  |
|  | *P (same)* | 0.2001 |  |
|  | *P* (bonf) | 0.2048 |  |
|  | Perm N | 9999 |  |

**
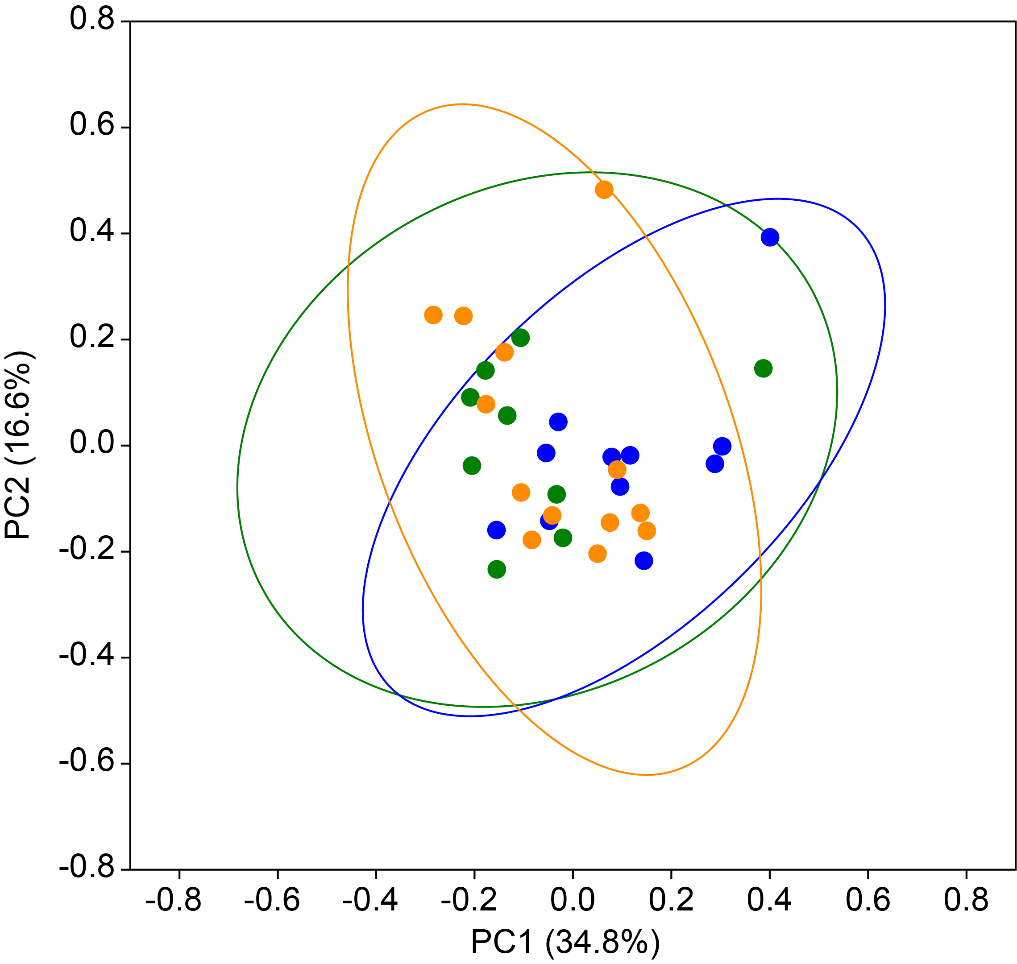
**

**Fig. S2** Principal Coordinates Analysis (PCoA) plot depicting compositional differences in predicted functional pathways across sample groups. Pathway abundances were derived from full-length 16S rRNA amplicon data analysed with PiCRUSt2. EC modules were aggregated prior to annotation to Level 3 hierarchical pathways. Relative abundances of pathways per sample were calculated, and Bray-Curtis dissimilarity was used as the distance metric for PCoA.

|  | **Table S10** Ordination analyses to describe microbiota and functional variation within the whole microbiota, core, and satellite taxa across chronic pancreatitis patients explained by significant clinical variables isolated from the forward stepwise-selection model. | | | | | | | | | | | | | | |  |
| --- | --- | --- | --- | --- | --- | --- | --- | --- | --- | --- | --- | --- | --- | --- | --- | --- |
|  |  |  |  |  |  |  |  |  |  |  |  |  |  |  |  |  |
| Structure (CCA) |  | Whole microbiota | | | |  | Core taxa | | | |  | Satellite taxa | | | |  |
|  |  | Contribution (%) | pseudo-*F* | *P* | *P* (adj) |  | Contribution (%) | pseudo-*F* | *P* | *P* (adj) |  | Contribution (%) | pseudo-*F* | *P* | *P* (adj) |  |
|  | Group |  |  |  |  |  | 37.8 | 1.7 | 0.002 | 0.008 |  | 29.3 | 1.3 | 0.001 | 0.004 |  |
|  | **Total** |  |  |  |  |  | **37.8** |  |  |  |  | **29.3** |  |  |  |  |
| Function (RDA) |  | Whole microbiota | | | |  | Core taxa | | | |  | Satellite taxa | | | |  |
|  |  | Contribution (%) | pseudo-*F* | *P* | *P* (adj) |  | Contribution (%) | pseudo-*F* | *P* | *P* (adj) |  | Contribution (%) | pseudo-*F* | *P* | *P* (adj) |  |
|  | Group |  |  |  |  |  | 63.2 | 4.1 | 0.002 | 0.009 |  |  |  |  |  |  |
|  | **Total** |  |  |  |  |  | **63.2** |  |  |  |  |  |  |  |  |  |
|  | Contribution (%) represents the percentage of the microbiota variation explained by a given variable with the respective analysis model. *P* (adj) is the adjusted significance value following false discovery correction. Group represents either diet-induced chronic pancreatitis (alcohol), or genetically related chronic pancreatitis (CFRP). CCA; Canonical correspondence analysis, RDA; Redundancy analysis. Pancreatic complications is an umbrella term to describe the presence of various manifestations, including pancreatic necrosis, presence of pseudocysts, and pancreatic ductal stricture. Missing data represents microbiota partitions for which the clinical variables did not significantly explain variation and can be found in Table S11. | | | | | | | | | | | | | | |  |

|  | **Table S11**. Non-significant clinical variables isolated from forward stepwise-selection ordination models to explain variance across microbiota structure and function across the whole, microbiota, core, and satellite taxa. | | | | | | | | | | | | | | |  |
| --- | --- | --- | --- | --- | --- | --- | --- | --- | --- | --- | --- | --- | --- | --- | --- | --- |
|  |  |  |  |  |  |  |  |  |  |  |  |  |  |  |  |  |
|  |  | **Whole microbiota** | | | |  | **Core taxa** | | | |  | **Satellite taxa** | | | |  |
|  |  | Contribution (%) | pseudo-*F* | *P* | *P* (adj) |  | Contribution (%) | pseudo-*F* | *P* | *P* (adj) |  | Contribution (%) | pseudo-*F* | *P* | *P* (adj) |  |
| **Structure (CCA)** | Age^†^ | 15.0 | 0.8 | 0.971 | 1.000 |  |  |  |  |  |  |  |  |  |  |  |
|  | BMI^†^ | 19.7 | 1.0 | 0.465 | 1.000 |  | 19.4 | 0.9 | 0.683 | 1.000 |  |  |  |  |  |  |
|  | Group* | 21.9 | 1.1 | 0.083 | 0.415 |  |  |  |  |  |  |  | | | |  |
|  | Pancreatic complications | 18.2 | 0.9 | 0.716 | 1.000 |  | 18.9 | 0.8 | 0.726 | 1.000 |  | 24.7 | 1.1 | 0.193 | 0.772 |  |
|  | Sex | 24.8 | 1.3 | 0.109 | 0.546 |  | 18.6 | 0.8 | 0.627 | 1.000 |  | 27.6 | 1.2 | 0.078 | 0.312 |  |
|  |  |  |  |  |  |  |  |  |  |  |  |  |  |  |  |  |
|  |  | **Whole microbiota** | | | |  | **Core taxa** | | | |  | **Satellite taxa** | | | |  |
|  |  | Contribution (%) | pseudo-*F* | *P* | *P* (adj) |  | Contribution (%) | pseudo-*F* | *P* | *P* (adj) |  | Contribution (%) | pseudo-*F* | *P* | *P* (adj) |  |
| **Function (RDA)** | Age | 26.7 | 1.8 | 0.084 | 0.505 |  | 9.3 | 0.5 | 0.793 | 1.000 |  | 12.3 | 0.7 | 0.6639 | 1.000 |  |
|  | BMI | 11.7 | 0.8 | 0.605 | 1.000 |  | 11.5 | 0.7 | 0.641 | 1.000 |  | 12.8 | 0.8 | 0.6203 | 1.000 |  |
|  | Group* | 29.0 | 2.0 | 0.059 | 0.355 |  |  |  |  |  |  | 23.0 | 1.4 | 0.1512 | 0.907 |  |
|  | Pancreatic complications^†^ | 9.1 | 0.6 | 0.797 | 1.000 |  |  |  |  |  |  | 21.8 | 1.4 | 0.1835 | 1.000 |  |
|  | Sex^†^ | 14.1 | 0.9 | 0.481 | 1.000 |  |  |  |  |  |  | 19.9 | 1.2 | 0.2446 | 1.000 |  |
|  | Contribution (%) represents the percentage of the microbiota variation explained by a given variable with the respective analysis model. *P* (adj) is the adjusted significance value following false discovery correction. Group represents either diet-induced chronic pancreatitis (alcohol), or genetically related chronic pancreatitis (CFRP). CCA; Canonical correspondence analysis, RDA; Redundancy analysis. Pancreatic complications is an umbrella term to describe the presence of various manifestations, including pancreatic necrosis, presence of pseudocysts, and pancreatic ductal stricture. *Missing values are variables identified as significant found in Table S9. ^†^Missing values are variables that were excluded from stepwise-selection model at origin (*P* = 1). | | | | | | | | | | | | | | |  |
|  |  |  |  |  |  |  |  |  |  |  |  |  |  |  |  |  |
|  |  |  |  |  |  |  |  |  |  |  |  |  |  |  |  |  |
|  |  |  |  |  |  |  |  |  |  |  |  |  |  |  |  |  |
|  |  |  |  |  |  |  |  |  |  |  |  |  |  |  |  |  |

| **Table S12**. Summary statistics for KEGG level 3 hierarchical pathway comparisons across CLR-transformed relative abundance data. | | | | | | | | | | | | |
| --- | --- | --- | --- | --- | --- | --- | --- | --- | --- | --- | --- | --- |
|  | **Group median CLR values** | | |  | **Kruskall-Wallis statistics** | | | |  | **Pairwise *p* (bonf):** | | |
| **Core taxa pathways (> 1%)** | **HC** | **AIP** | **CFRP** |  | ***Df*** | **KW-χ²** | ***p*** | ***p* (FDR)** |  | **AIP vs HC** | **CFRP vs HC** | **AIP vs CFRP** |
| ABC transporters | 0.0157 | -0.0214 | 0.0076 |  | 2 | 1.3602 | 0.5066 | 0.5741 |  | 1.0000 | 1.0000 | 0.9300 |
| Amino sugar and nucleotide sugar metabolism | -0.0287 | 0.0341 | -0.0013 |  | 2 | 8.2972 | 0.0158 | 0.2404 |  | **0.0350** | 1.0000 | 0.0730 |
| Biosynthesis of amino acids | 0.0361 | -0.0080 | 0.0155 |  | 2 | 3.8060 | 0.1491 | 0.3860 |  | 0.1720 | 1.0000 | 0.7050 |
| Biosynthesis of cofactors | -0.0015 | 0.0044 | -0.0076 |  | 2 | 0.1091 | 0.9469 | 0.9469 |  | 1.0000 | 1.0000 | 1.0000 |
| Biosynthesis of secondary metabolites | 0.0154 | -0.0051 | 0.0029 |  | 2 | 3.3461 | 0.1877 | 0.3860 |  | 0.2020 | 0.9720 | 1.0000 |
| Carbon metabolism | 0.0030 | -0.0012 | -0.0053 |  | 2 | 2.7360 | 0.2546 | 0.4162 |  | 1.0000 | 0.2990 | 1.0000 |
| Cysteine and methionine metabolism | 0.0280 | -0.0287 | 0.0094 |  | 2 | 4.5712 | 0.1017 | 0.3860 |  | 0.1280 | 1.0000 | 0.4380 |
| Glycolysis / Gluconeogenesis | -0.0118 | -0.0026 | 0.0156 |  | 2 | 4.0463 | 0.1322 | 0.3860 |  | 0.5010 | 0.2100 | 1.0000 |
| Metabolic pathways | -0.0066 | -0.0008 | -0.0008 |  | 2 | 2.0137 | 0.3654 | 0.4778 |  | 0.4980 | 1.0000 | 1.0000 |
| Microbial metabolism in diverse environments | -0.0175 | 0.0022 | -0.0076 |  | 2 | 0.9994 | 0.6067 | 0.6446 |  | 0.9660 | 1.0000 | 1.0000 |
| Nucleotide metabolism | -0.0028 | -0.0111 | 0.0055 |  | 2 | 1.3932 | 0.4983 | 0.5741 |  | 1.0000 | 1.0000 | 0.7230 |
| Purine metabolism | -0.0009 | -0.0078 | 0.0060 |  | 2 | 3.1760 | 0.2043 | 0.3860 |  | 1.0000 | 1.0000 | 0.2730 |
| Pyrimidine metabolism | 0.0037 | -0.0053 | 0.0037 |  | 2 | 4.1039 | 0.1285 | 0.3860 |  | 0.3690 | 1.0000 | 0.2030 |
| Quorum sensing | 0.0318 | -0.0579 | 0.0101 |  | 2 | 2.4496 | 0.2938 | 0.4162 |  | 0.3030 | 1.0000 | 1.0000 |
| Ribosome | 0.0449 | -0.0171 | 0.0168 |  | 2 | 7.1313 | 0.0283 | 0.2404 |  | **0.0130** | 1.0000 | 0.4170 |
| Starch and sucrose metabolism | -0.0193 | -0.0355 | 0.0432 |  | 2 | 3.6282 | 0.1630 | 0.3860 |  | 1.0000 | 0.2840 | 0.4170 |
| Two-component system | -0.0289 | 0.0391 | 0.0169 |  | 2 | 2.4968 | 0.2870 | 0.4162 |  | 1.0000 | 1.0000 | 0.3510 |
|  | **Group median CLR values** | | |  | **Kruskall-Wallis statistics** | | | |  | **Pairwise *p* (bonf):** | | |
| **Satellite taxa pathways (> 1%)** | **HC** | **AIP** | **CFRP** |  | ***Df*** | **KW-χ²** | ***p*** | ***p* (FDR)** |  | **AIP vs HC** | **CFRP vs HC** | **AIP vs CFRP** |
| ABC transporters | -0.0170 | -0.0544 | 0.0689 |  | 2 | 10.1368 | 0.0063 | **0.0493** |  | 1.0000 | 0.0760 | **0.0050** |
| Amino sugar and nucleotide sugar metabolism | -0.0631 | 0.0255 | -0.0109 |  | 2 | 5.8824 | 0.0528 | 0.1584 |  | 0.0930 | 0.1820 | 1.0000 |
| Aminoacyl-tRNA biosynthesis | 0.0177 | 0.0170 | -0.0098 |  | 2 | 1.4976 | 0.4729 | 0.6081 |  | 1.0000 | 0.4920 | 1.0000 |
| Biosynthesis of amino acids | -0.0016 | -0.0003 | -0.0079 |  | 2 | 0.6037 | 0.7394 | 0.7722 |  | 1.0000 | 1.0000 | 1.0000 |
| Biosynthesis of cofactors | 0.0332 | -0.0019 | 0.0154 |  | 2 | 3.5204 | 0.1720 | 0.3420 |  | 0.3930 | 0.2870 | 1.0000 |
| Biosynthesis of secondary metabolites | 0.0111 | -0.0098 | 0.0043 |  | 2 | 3.5366 | 0.1706 | 0.3420 |  | 0.2020 | 0.9720 | 1.0000 |
| Carbon metabolism | -0.0136 | 0.0067 | 0.0001 |  | 2 | 5.2743 | 0.0716 | 0.1840 |  | **0.0380** | 0.9720 | 0.9090 |
| Cysteine and methionine metabolism | 0.0170 | 0.0084 | -0.0031 |  | 2 | 1.8100 | 0.4046 | 0.5601 |  | 1.0000 | 0.5580 | 1.0000 |
| Glycolysis / Gluconeogenesis | -0.0488 | 0.0299 | -0.0046 |  | 2 | 9.6014 | 0.0082 | **0.0493** |  | **0.0170** | **0.0270** | 0.9930 |
| Metabolic pathways | 0.0001 | -0.0012 | 0.0034 |  | 2 | 0.6443 | 0.7246 | 0.7722 |  | 1.0000 | 0.9720 | 1.0000 |
| Microbial metabolism in diverse environments | -0.0124 | 0.0023 | 0.0030 |  | 2 | 2.7652 | 0.2509 | 0.4106 |  | 0.4560 | 0.4920 | 1.0000 |
| Nucleotide metabolism | 0.0081 | -0.0037 | 0.0034 |  | 2 | 0.5171 | 0.7722 | 0.7722 |  | 1.0000 | 1.0000 | 1.0000 |
| Purine metabolism | 0.0033 | -0.0015 | 0.0217 |  | 2 | 3.3214 | 0.1900 | 0.3420 |  | 0.7830 | 1.0000 | 0.2460 |
| Pyrimidine metabolism | 0.0218 | -0.0050 | 0.0023 |  | 2 | 2.1153 | 0.3473 | 0.5209 |  | 0.6030 | 0.7860 | 1.0000 |
| Quorum sensing | -0.0282 | -0.0476 | 0.0536 |  | 2 | 11.8739 | 0.0026 | **0.0475** |  | 1.0000 | **0.0060** | **0.0120** |
| Ribosome | 0.0374 | -0.0047 | -0.0203 |  | 2 | 8.4253 | 0.0148 | 0.0570 |  | 0.5250 | **0.0060** | 0.6210 |
| Starch and sucrose metabolism | -0.1207 | 0.0715 | 0.0550 |  | 2 | 8.2921 | 0.0158 | 0.0570 |  | 0.0930 | **0.0100** | 1.0000 |
| Two-component system | 0.0213 | -0.0111 | -0.0137 |  | 2 | 1.1046 | 0.5756 | 0.6908 |  | 1.0000 | 1.0000 | 1.0000 |
|  | **Group median CLR values** | | |  | **Kruskall-Wallis statistics** | | | |  | **Pairwise *p* (bonf):** | | |
| **Satellite taxa exclusive GI-pathways** | **HC** | **AIP** | **CFRP** |  | ***Df*** | **KW-χ²** | ***p*** | ***p* (FDR)** |  | **AIP vs HC** | **CFRP vs HC** | **AIP vs CFRP** |
| Alcoholism | 2.7292 | 1.4464 | 0.3005 |  | 2 | 0.9416 | 0.6245 | 0.6833 |  | 1.0000 | 1.0000 | 1.0000 |
| Biosynthesis of various alkaloids | -0.7538 | -0.7538 | -0.7538 |  | 2 | 1.8224 | 0.4020 | 0.6551 |  | 0.6450 | 1.0000 | 1.0000 |
| Biosynthesis of various other secondary metabolites | 0.1452 | 1.2345 | 2.1885 |  | 2 | 3.1965 | 0.2023 | 0.6068 |  | 0.4800 | 0.4530 | 1.0000 |
| *Bisphenol degradation | -0.2385 | -0.2385 | -0.2385 |  | 2 | 2.0000 | 0.3679 | 0.6551 |  | NA | NA | NA |
| Caffeine metabolism | -0.9597 | -0.9597 | -0.9597 |  | 2 | 1.6568 | 0.4367 | 0.6551 |  | 0.9450 | 1.0000 | 0.6150 |
| Colorectal cancer | 3.0256 | 1.5059 | -0.7306 |  | 2 | 8.0573 | 0.0178 | 0.1476 |  | 0.2220 | 0.0280 | 0.5100 |
| Non-alcoholic fatty liver disease | 2.8568 | 1.4484 | -0.7688 |  | 2 | 6.8350 | 0.0328 | 0.1476 |  | 0.3060 | 0.0560 | 0.5430 |
| Polycyclic aromatic hydrocarbon degradation | 2.3212 | 0.7711 | 1.3525 |  | 2 | 0.7618 | 0.6833 | 0.6833 |  | 1.0000 | 0.8970 | 1.0000 |
| Vibrio cholerae infection | 3.1639 | -4.5984 | 1.7776 |  | 2 | 1.1423 | 0.5649 | 0.6833 |  | 0.9900 | 1.0000 | 1.0000 |

Given the instability of normality across CLR-transformed table features (core; 64.7%, sat; 33.3%, sat exclusive GI; 0%), Kruskal-Wallis testing was utilised to assess overall group differences. Subsequent Mann-Whitney U testing was then applied to investigate specific pairwise-differences across individual features with Bonferroni correction applied.


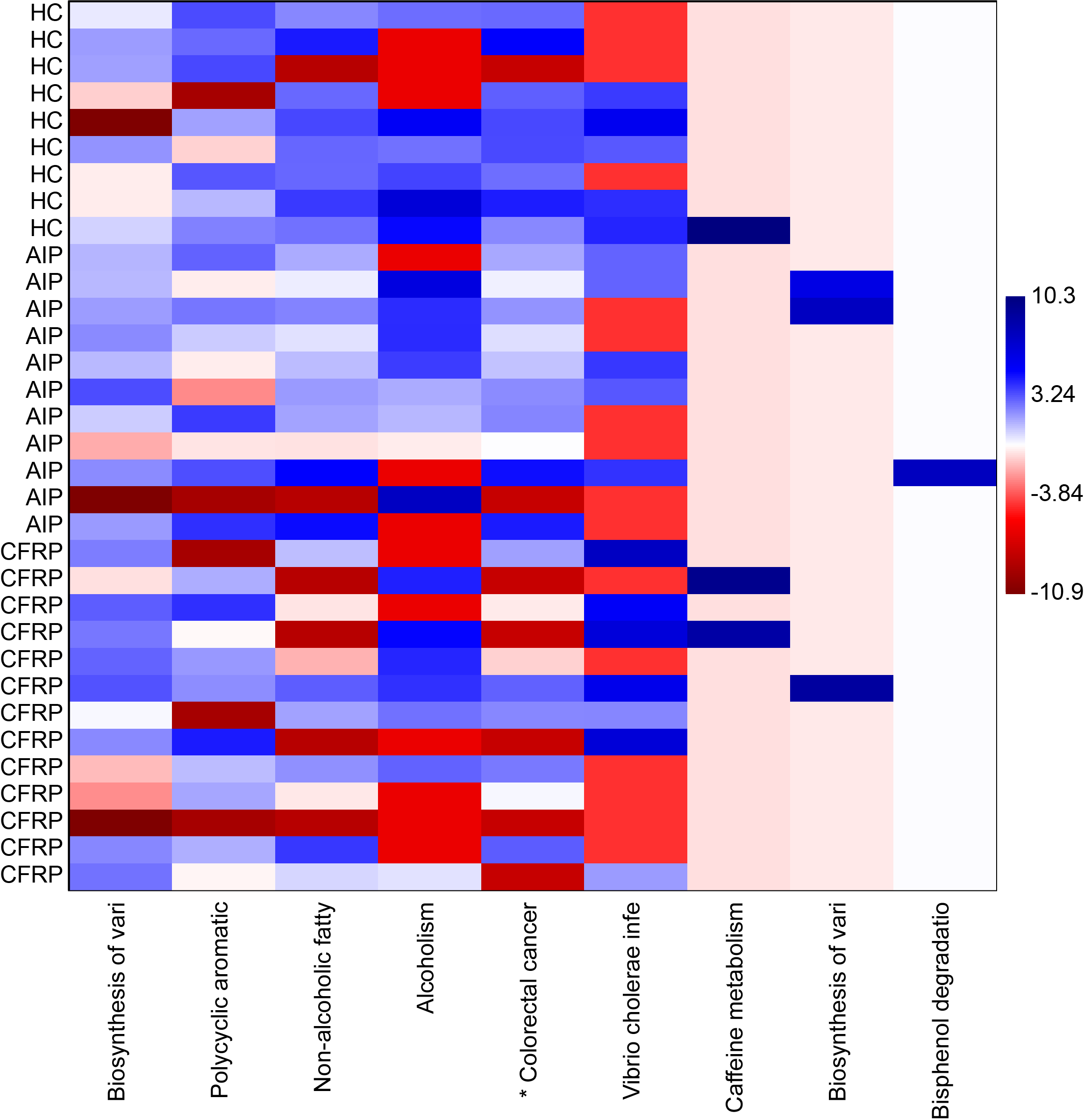


**Fig. S3** Differences in predicted metagenome function exclusive to the satellite taxa between study groups. CLR transformed compositional data was plotted to visualise differences among groups across KEGG level 3 hierarchical pathways relating to GI functionality and related physiological processes. Asterisks (*) denote significant differences across group means following ANOVA testing (*P* < 0.05). Full length pathway names and associated summary statistics can be found in Tables S12-S13.

| **Table S13** Details of KEGG level 3 hierarchical pathways. | |
| --- | --- |
| **Abbreviation** | **Full pathway name** |
| Amino sugar and nucl. | Amino sugar and nucleotide sugar metabolism |
| Aminoacyl-tRNA biosy. | Aminoacyl-tRNA biosynthesis |
| Biosynthesis of amin. | Biosynthesis of amino acids |
| Biosynthesis of cofa. | Biosynthesis of cofactors |
| Biosynthesis of seco. | Biosynthesis of secondary metabolites |
| Biosynthesis of vari. | Biosynthesis of various other secondary metabolites |
| Biosynthesis of vari. | Biosynthesis of various alkaloids |
| Bisphenol degredatio. | Bisphenol degradation |
| Cysteine and methion. | Cysteine and methionine metabolism |
| Glycolysis / Glucone. | Glycolysis / Gluconeogenesis |
| Microbial metabolism. | Microbial metabolism in diverse environments |
| Non-alcoholic fatty. | Non-alcoholic fatty liver disease |
| Nucleotide metabolis. | Nucleotide metabolism |
| Polycyclic aromatic. | Polycyclic aromatic hydrocarbon degradation |
| Pyrimidine metabolis. | Pyrimidine metabolism |
| Starch and sucrose m. | Starch and sucrose metabolism |
| Vibrio cholerae infe. | Vibrio cholerae infection |
